# Mental health matters: Evaluating the preparedness of sport psychologists to incorporate within their role

**DOI:** 10.1002/ejsc.12205

**Published:** 2024-10-27

**Authors:** Stacy Winter, Dave Collins

**Affiliations:** ^1^ School of Sport, Exercise and Applied Science, St Mary's University London UK; ^2^ Moray House School of Education and Sport, The University of Edinburgh Edinburgh UK; ^3^ Grey Matters Performance Ltd UK

**Keywords:** behavior implementation, theoretical domains framework, well‐being

## Abstract

Mental health has become increasingly important for the applied sport psychologist, a factor which may include screening, promoting literacy, individual support, and educational programs. However, despite of this importance, few checks have been made on the perceived preparedness of sport psychologists to undertake this work and whether differences may exist between those recently qualified compared to experienced practitioners. Underpinned by the theoretical domains framework, 62 Health and Care Professions Council registered practitioner sport and exercise psychologists (30 females: age: *M* = 46.13 years and SD = 10.44 years and 32 males: age: *M* = 43.25 years and SD = 10.47 years) completed an online survey to assess whether they felt equipped to address the mental health dimension of their work. The survey comprised three sections: Demographic and background information, a series of statements (*n* = 31) adapted from the determinants of implementation behavior questionnaire, and three questions with space for free text comments, inviting participants to explain their level of preparedness to incorporate and deliver mental health interventions. A 6 × 12 analyses of variance yielded significant differences between domain ratings but not across levels of experience or interactions. Data from free text comments were analyzed thematically and categorized into the following three themes: (a) complementing the performance role, (b) awareness of professional boundaries, and (c) importance of further continued professional development. Combined, although practitioners emphasized importance, preparedness ratings ranged from neutral to somewhat agree, calling for further specific sport psychology‐based mental health training.

## Highlights

- An online survey, underpinned by the theoretical domains framework, was used to assess whether registered sport and exercise psychology practitioners in the United Kingdom felt prepared to undertake the mental health dimension of their work.
- Analysis through ANOVA yielded no significant differences across experience or interactions, suggesting that preparedness and implementation do not differ regardless of whether recently qualified or experienced practitioners. Furthermore, average ratings across all domains revealed practitioners feel only “somewhat prepared” to address client needs in this area.
- Free text comments provided additional insight to levels of preparedness where practitioners noted the frequent necessity to offer mental health support, why it complements the performance focus, and how they are uniquely placed to understand the specific contextual demands, yet were cognizant of their competencies, knowledge base, and boundaries.
- Calls for further specific sport psychology‐based mental health training are warranted by the professional bodies in the United Kingdom to ensure practitioners feel equipped to address this increasingly important factor of their work.

## 1 INTRODUCTION

As defined by the World Health Organization (WHO, 2001), mental health is a state of well‐being in which every individual realizes their own potential, can cope with the normal stresses of life, can work productively and fruitfully, and is able to contribute to her or his community. Schinke et al. (2018) conceptualized mental health on a continuum, with one end representing high‐functioning individuals whose psychological states do not interfere with daily activities and the other representing low functioning individuals whose psychological states consist of a variety of problematic cognitive, emotional, or behavioral characteristics, often referred to as mental illness. This continuum thus allows for an appreciation that, between the two‐continuum ends, lie degrees of psychological wellness/distress and effective/reduced functioning.

Although mental health has always been a concern for psychologists working in sport, this has recently become an even more emphasized feature of their work (Vella et al., 2021). Examples of mental health work for the practicing sport psychologist include but are not limited to screening, promoting literacy, individual support, and educational programs. Position statements (e.g., Reardon et al., 2019) have focused on this, whereas researchers (e.g., Collins & Winter 2020) have begun to offer models of practice for the incorporation of mental health into the sport psychologist's work portfolio. However, while the importance of mental health is accepted and demonstrated by its incorporation within both current sport psychology roles and position descriptions in job advertisements, formal training has only recently been made an explicit requirement of training programs in the United Kingdom. For example, the British Association of Sport and Exercise Sciences (BASES) sport and exercise psychology accreditation route (SEPAR) which now includes mental health units. As such, we were interested to ascertain the extent to which practitioners, many of whom may not have completed formal training on this factor, felt equipped to address mental health. Accordingly, we looked for research in similar areas which had examined the incorporation of new responsibilities for already accredited professionals.

A parallel challenge was considered in the work of McParlin et al. (2017), who examined the willingness and preparedness of midwives to prescribe exercise interventions for obese pregnant women. In simple terms, this required well‐trained specialists to operate outside of their direct expertise to address an important adjunct issue. McParlin et al. (2017) used the theoretical domains framework (TDF; Michie et al., 2005) to examine the attitudes of practicing midwives. The TDF is a framework, which provides a theoretical lens through which to view cognitive, affective, social, and environmental influences on behavior (Atkins et al., 2017). The TDF was developed to evaluate the implementation of guidelines by health care professionals, particularly where such guidelines might be seen to take the professional outside of his or her initially trained “comfort zone”. The approach uses umbrella terms relating to various theoretical approaches of evidence‐based practice and aids understanding of the key domains associated with behavior implementation. Specifically, the TDF was derived from 33 theories of behavior, with the resulting framework originally identifying 12 theoretically distinct domains (McGowan et al., 2020). Huijg et al. (2014) developed the determinants of implementation behavior questionnaire (DIBQ) as a valid and reliable measure following the 12 theoretically distinct domains of the TDF. The authors reported confirmatory factor analysis and Cronbach's alpha demonstrated high internal consistency reliability, with values ranging from 0.68 to 0.93, respectively.

Therefore, using this methodology with the requisite modifications, we sought to ascertain the extent to which sport psychology professionals perceived themselves to be adequately equipped to address the mental health dimension of their work. Furthermore, given its recent impetus, whether more experienced or those recently qualified individuals would perceive greater preparedness. Based on our own affiliation and specific country guidelines, our investigation was delimited to the UK setting.

## 2 METHOD

### 2.1 Participants

Following institutional ethical approval, individuals were contacted via direct email, social media posts, and professional association channels (e.g., BASES communicated to all their psychology division members) inviting them to participate in the study. Our inclusion criteria stipulated current registration as a sport and exercise psychologist with the Health and Care Professions Council (HCPC), the statutory regulator for practitioner psychologists in the United Kingdom. Subsequently, 62 HCPC registered sport and exercise psychologists were recruited to participate in the study. The sample comprised 30 females (age: *M* = 46.13 years and SD = 10.44 years) and 32 males (age: *M* = 43.25 years and SD = 10.47 years). Collectively, a number of different nationalities were reported. We have purposely kept this information confidential, in order to protect participant anonymity in compliance with the general data protection regulation (GDPR). Additionally, in accordance with GDPR, the HCPC do not publish how many practitioners are currently registered as sport and exercise psychologists within the United Kingdom. Based on previously released data, and with allowance made for recent growth, we estimated the current number of registered individuals at c500. As such, our sample reflected around 12% of the current estimated population.

### 2.2 Design

A mixed‐method explanatory design (Creswell & Plano Clark, 2011) was used whereby the secondary component elicits a viewpoint that the initial results cannot reach. In this study, the qualitative component of the survey explained the data built from the DIBQ quantitative findings to allow for additional insights in terms of sport psychologist's preparedness to incorporate mental health.

### 2.3 Measures

The survey was designed in three sections and commenced with demographic questions and background information regarding the respondent's age, nationality, gender identify with, any professional qualifications held in addition to HCPC registration, number of years practicing (since qualified), sport/s currently working in, and whether completed any mental health training. In the second section, a series of statements (*n* = 31) were adapted from the DIBQ (Huijg et al., 2014) to ensure relevance for the present study (e.g., “physical activity” was changed to “mental health”). DIBQ is based on the 12‐domain original version of the TDF (Michie et al., 2005) and was used to establish the factors influencing participant choice to promote mental health or not (see Table 1). In‐line with Kunstler et al. (2019), each of the 12 behavioral domains contributed between two and six questions and were assorted randomly in the survey. Respondents indicated the extent to which they agreed/disagreed with each statement on a five‐point Likert scale (1 = “*strongly disagree*” and 5 = “*strongly agree*”). Three items required the Likert scale to be amended so the statement aligned to appropriate responses. For example, a statement from the beliefs about consequences TDF domain: “For me, delivering mental health interventions is…” was amended to 1 = “*not worthwhile at all*”, 5 = “*very worthwhile*.”

**TABLE 1:** Description of the behavioral domains and summary statistics (*n* = 62).

Domain	Description	Mean (SD)
1. Knowledge	Do practitioners know what to advise athletes regarding their mental health?	3.89 (0.91)
2. Skills	Do practitioners feel able and have the correct training to advise athletes about mental health?	3.33 (0.83)
3. Professional role	Do practitioners feel advising athletes about mental health is part of their professional responsibility?	3.63 (0.94)
4. Capabilities	Do practitioners feel capable, confident, and comfortable to advise athletes about mental health?	3.35 (0.68)
5. Consequences	What do practitioners think will be the result if they advise athletes about mental health?	3.88 (0.41)
6. Motivation and goals	How much do practitioners aim to or want to discuss and advise athletes about mental health?	3.52 (0.79)
7. Decision and attention	Do practitioners remember to discuss or remember that they should discuss mental health with athletes?	3.38 (0.84)
8. Social influences	Do other professionals or individuals influence whether practitioners discuss and advise athletes about mental health?	3.51 (0.56)
9. Emotion	Do emotions/feelings influence whether practitioners discuss and advise athletes about mental health?	3.19 (0.49)
10. Behavior regulation	Are there guidelines in place to support practitioners when they discuss mental health with athletes?	3.92 (0.63)
11. Environment and resources	Do practitioners have enough resources, such as time, leaflets, and referral options, to discuss mental health with athletes?	3.22 (0.72)
12. Nature of the behavior	Do practitioners discuss mental health with athletes and advise them in accordance with published guidelines?	3.50 (0.87)

In the final section, we probed what influenced these practicing sport psychologists' responses. Participants were posed three questions with space for free text comments: (1) If you feel prepared to incorporate and deliver mental health interventions within your applied practice, please state why? (2) If you feel unprepared, please tell us why? (3) To be more prepared, what do you feel you would need?’ By including these open questions, we hoped to learn what participants thought and felt about giving mental health advice, enabling some guidance or insight into why implementation might vary (Michie et al., 2005).

### 2.4 Procedure

A preliminary pilot study was conducted with six registered sport psychologists to ensure that questions and format of the survey were clear and understandable. Using a cognitive interviewing process (Collins & Winter 2020), it was collectively agreed that a clear definition and examples of mental health interventions be provided for clarity. Thus, at the start of section two on implementation, we drew upon the WHO (2001) definition and Schinke et al. (2018) continuum conceptualization of mental health and stated the following: examples of mental health interventions include but are not limited to screening, promoting literacy, individual support, and educational programs.

Following these amendments, the final version was released online via joint information systems committee (JISC) online surveys. Participants were initially invited to read through the information sheet and, if happy to proceed, were presented with the consent form to complete before the survey commenced. Respondents were advised that all data collected would be used for the sole purpose of the research project and only members of the research team would have access to the information. Following completion of section one, all participants followed a standardized procedure. They were asked to consider a number of statements regarding their engagement with mental health in their applied sport psychology work, by selecting the appropriate number from the five‐point Likert scale. We stressed that there were no right or wrong answers, we just wanted to know about their experience.

### 2.5 Data analysis

Quantitative data were analyzed through the use of SPSS (version 26). Specifically, we looked at the level of perceived preparedness (*Means* and *SD*) against different categories, then completed a 6 × 12 (Experience × Domain) ANOVA with repeated measures on the second factor. Free text comments were retrieved from JISC and reviewed by the authors, experienced as both qualitative researchers and HCPC registered practitioner psychologists. These comments were then analyzed thematically by the first author, following Braun and Clarke's (2006) six‐step protocol to construct key points of interest that were interpreted as meaningful and relevant to the study. The coauthor acted as a critical friend (Smith & McGannon, 2018) throughout, who provided different plausible interpretations and served to encourage further reflection. The authors debated coherence and distinctiveness from one theme to another to ensure meaningful representations of the data (Braun et al., 2017).

## 3 RESULTS

In addition to HCPC registration, 22 participants also reported being BASES accredited practitioners and 24 participants held British Psychological Society (BPS) chartered psychologist status. BASES and BPS govern the two professional training routes within the United Kingdom (SEPAR and qualification in sport and exercise psychology; QSEP Stage 2). Successful completion of these routes confers eligibility to apply for registration with the HCPC. Participants stated the following number of years’ experience practicing as a registered sport and exercise psychology practitioner: 0–5 years (38.7%), 6–10 years (9.68%), 11–15 years (9.68%), 16–20 years (19.35%), 21–25 years (12.9%), and more than 25 years (9.68%). Participants' experiences ranged from working full‐time with elite performers via an organizational body or their own private practices, through to consulting with a variety of professional (e.g., golf, football, and motorsport), Olympic (e.g., gymnastics, triathlon, and rowing), and disability sports (e.g., wheelchair rugby, wheelchair tennis, and para‐athletics).

With regards to additional training, 34 participants (54.84%) had completed mental health first aid (MHFA) and five participants (8.06%) the SEPAR mental health units. Seven (11.29%) had received counseling training, with one practitioner also chartered as a counseling psychologist. Five respondents were trained in suicide awareness and eating disorders, respectively. Finally, seven reported no formal/certified course under the mental health banner but saw this as an inherent part of continued professional development (CPD) activities, whereas 16 individuals (25.64%) stated they had received no mental health training.

Table 1 presents the summary statistics for participant data, together with descriptions of the different domains based on McParlin et al. (2017). Table 2 presents the mean and standard deviations for the 12 domains across the six experience categories. Analysis through ANOVA yielded significant differences between domain ratings (F_(7.6, 426)_ = 6.99 and *p* < 0.001). Greenhouse–Geisser adjustments were applied following a significant Mauchly’s spericity test and the effect size was 0.11 or small (cf. Cohen, 1988). Follow‐up using Tukey’s test showed this to be due to differences between the high mean scores of behavior regulation and knowledge (3.92 and 3.89, respectively) against the lower end of emotion and environment and resources (3.19 and 3.22). No significant difference emerged for experience nor for the domain by experience interaction (*p* > 0.05; ES = 0.65 and 0.62, respectively—both small). Notably across all domains, practitioners rated their average levels of preparedness as neutral to somewhat agree (equivalent to Likert scores of 3–4, respectively,) regardless of experience.

**TABLE 2:** Mean (SD) scores for behavioral domains across experience categories (*n* per category as shown).

	0–5 years (*n* = 24)	6–10 years (*n* = 6)	11–15 years (*n* = 6)	16–20 years (*n* = 12)	21–25 years (*n* = 8)	25+ yrs (*n* = 6)
1. Knowledge	3.8 (1.0)	4.1 (0.66)	3.6 (0.66)	4.3 (0.68)	3.7 (1.2)	3.7 (0.76)
2. Skills	3.2 (0.81)	3.4 (0.86)	3.6 (0.68)	3.4 (0.66)	3.4 (1.2)	2.9 (0.91)
3. Professional role	3.5 (0.97)	3.6 (0.81)	3.0 (0.63)	4.1 (0.99)	3.5 (0.76)	3.8 (1.2)
4. Capabilities	3.3 (0.66)	3.2 (0.98)	3.3 (0.60)	3.6 (0.61)	3.2 (0.84)	3.2 (0.52)
5. Consequences	3.8 (0.36)	4.0 (0.43)	4.3 (0.28)	3.8 (0.39)	4.0 (0.50)	3.6 (0.33)
6. Motivation and goals	3.5 (0.85)	3.9 (0.49)	3.9 (0.58)	3.6 (0.83)	3.2 (0.80)	2.9 (0.73)
7. Decision and attention	3.38 (0.89)	3.2 (0.98)	3.4 (0.86)	3.7 (0.78)	3.2 (0.59)	3.1 (0.91)
8. Social influences	3.5 (0.59)	3.4 (0.64)	3.4 (0.62)	3.6 (0.59)	3.5 (0.56)	3.6 (0.49)
9. Emotion	3.2 (0.55)	3.4 (0.48)	3.2 (0.58)	3.1 (0.43)	3.0 (0.38)	3.3 (0.46)
10. Behavior regulation	3.8 (0.79)	3.9 (0.50)	3.7 (0.49)	4.2 (0.54)	4.0 (0.50)	4.1 (0.25)
11. Environment and resources	3.1 (0.86)	3.4 (0.62)	3.0 (0.52)	3.2 (0.64)	3.4 (0.86)	3.2 (0.45)
12. Nature of the behavior	3.58 (0.92)	3.6 (0.38)	3.5 (1.0)	3.5 (0.73)	3.5 (1.0)	3.2 (1.0)

Of the 62 participants who completed the survey, 48 made at least one free text comment (77.42%). Following the thematic analysis process, data were categorized into three key themes: (a) complementing the performance role, (b) awareness of professional boundaries, and (c) importance of further CPD. Themes are presented below with representative verbatim quotes and participant identities protected through pseudonyms.

### 3.1 Complementing the performance role

Participants not only felt that mental health support was a part of their role but also complemented the performance environment: “For me, a ‘mentally healthy’ individual tends to be a more effective sports performer. Thus, I view providing mental health interventions complements the range of applied sport psychology delivery” (Sarah). From experience, Tom conveyed the frequent necessity he has incurred to offer mental health support: “I feel this is necessary as the majority of sportspeople I've worked with, seem to have challenges linked to poor mental health. In my opinion, these challenges should be addressed as a priority”. Regarding the interrelationship between performance and mental health support, Emily stated: “It comes naturally human being first, athlete second” (Emily). However, an interesting contrast was offered by this point from Simon: “I feel there is still a conflict between work on the ‘person’ and on the ‘athlete’ and this often causes a sense of incongruence within me as I have to take the lead of the athlete”. Thus, whether mental health is sometimes deemed outside of the performance “athlete” focus. Finally, Craig highlighted how sport psychology practitioners could be best placed to offer initial support:

As sport psychologists we are uniquely placed to understand the constant mental health challenges that elite sport creates everyday…Furthermore, we know that early intervention is really beneficial, so we are more likely to be able to spot problems early and address them in useful ways before athletes fall off the edge of the cliff.

### 3.2 Awareness of professional boundaries

Within this UK sample, sport psychology practitioners were cognizant of their competencies, knowledge base, and boundaries: “I'm not a clinical psych so I know there is a limit to my ability” (James). Being aware of this led practitioners to have referral services in place: “I would feel unprepared if the person was presenting with an issue that I was not trained in, I would feel prepared to sign post to an appropriate service” (Sally). That being said, the referral process also led to some feelings of unpreparedness: “Mental health covers a range of issues and includes serious clinical issues. The decision to refer on is key and that is why I feel under prepared at times” (Matthew).

Reasons why participants felt prepared to offer mental health support within their professional boundaries included specific training, following professional guidance, counseling skills, experience, and alignment with their philosophy as expressed by Lizzie: “Having attended (MHFA), while holding an undergraduate degree in psychology and specific CPD activities has supported me to feel more confident incorporating mental health interventions within practice”. Though she did go on to state: “This confidence is somewhat limited to environments within which I have a high contextual understanding”. Further, Hannah reported how she would like mental health training that would be better recognized within our boundaries by other professionals:

Sometimes there is a snobbery that as a sport and exercise psychologist we should stay in an education/mental skills lane, but some things could effectively allow us to provide recognized support (especially when we may be the client's only option i.e., there are long wait lists and limited mental health support available elsewhere).

### 3.3 Importance of further CPD

Unanimous across practitioners was the need to be proactive in keeping up to date with the mental health literature and engage in further training and CPD. Ultimately, this stemmed from: “There was no emphasis/literature on this when I trained” (Joe) and: “Don't feel I've had the training or knowledge from my education” (Anne). Participants identified potential limitations with the specific training routes to become a HCPC registered sport and exercise psychologist: “I don't feel that the training as sport psychologists in the UK take mental health as seriously as other psychological training, which leaves a lot of practitioners unknowingly down dangerous situations when working with athletes” (David). Building on from this, William questioned: “Have we got our ‘training structures’ in order or are we too siloed?” For practitioners to feel more prepared incorporating mental health support within their sport psychology roles, Lucy commented: “I feel I would need further training and experience and exposure to mental health contexts…Ultimately there needs to be ‘certified’ courses/programmes available for sport psychs”. Indeed, this was something one practitioner had actively engaged in:

I have had adequate and extra training that I have sought despite BASES and BPS not putting as much pressure to do this in my training. I have been working in mental health settings within the NHS and private health care part‐time as well as in sport, so I have immersed myself in mental health systems to learn about them to make my practice within sport better.

(Helen)

Suggestions to make practitioners feel more prepared included: “Greater clarification from professional codes of conduct what one can and can't do without clinical training” (Emma), “Sport psychology specific CPD focusing on delivering mental health interventions” (Laura), “Greater depth of understanding on the signs and symptoms of specific sub‐clinical and clinical issues” (Alex), “Access to more sport specific resources for athletic populations” (Stephen), and “More official training to ensure it fits into codes of conduct/competencies in order for me to do it” (Ian).

## 4 DISCUSSION

To our knowledge, this was the first study to investigate the extent to which sport psychology professionals felt equipped to address the mental health dimension of their work. Incorporating TDF (Michie et al., 2005) provided a structured approach to cover a broad range of constructs, minimizing the risk that influential factors were missed (Michie et al., 2007). Furthermore, TDF has been used by other health care professionals (e.g., Kunstler et al., 2019; McParlin et al., 2017) to investigate the implementation of evidence‐based guidelines using both qualitative and quantitative approaches, and therefore was deemed a suitable tool to investigate sport psychologists' behavior.

Our participants, all registered sport and exercise psychologists, verbalized the extent to which they deemed mental health was and should be an important dimension to their applied work (Reardon et al., 2019; Vella et al., 2021). Practitioners were able to draw upon their experiences and highlighted the frequent necessity to offer mental health support, why it complements the performance focus, and how they are uniquely placed to understand the specific contextual demands. However, notably, the domain results indicated that, as a group, the practitioners only rated their levels of preparedness as neutral to somewhat agree in terms of the belief that they hold the necessary knowledge, skills, and capabilities to incorporate and implement mental health support work.

With regards to the key constructs associated with behaviors, there were significant differences between the extremes on domain ratings suggesting that participants felt adequately prepared in terms of knowledge and professional guidelines but less so in terms of emotional and other resources. The lack of significance across experience or interactions suggests that preparedness and implementation do not differ regardless of whether practitioners were recently qualified or had accrued years of experience.

In comparison to McParlin et al. (2017), who examined the incorporation of new responsibilities (prescribing exercise interventions for obese pregnant women) for already accredited professionals (midwives), our practitioners reported identical beliefs about consequences (*M* = 3.88) and both ranked this domain third highest in their levels of preparedness. In other words, these areas were clearly seen as important but practitioners were only “somewhat certain” that their advice would carry positive consequences. There were also similarities to be found where practitioners felt less equipped in their implementation. This was demonstrated by both midwives and sport psychologists rating their skills and environment/resources as two of the lowest domains, although we recognize issues with generalizing across samples (Smith, 2018). In summary, mental health was clearly seen as important but our practitioners felt that their preparation to address this had room for improvement.

It is also worth noting that our investigation was delimited to the UK setting; an issue against country‐specific training routes and organizational guidelines. There is also a risk for selection bias, where practitioners specifically interested in mental health promotion might have been more inclined to complete the survey and therefore a response bias may have occurred. Furthermore, the initial section of the survey was reliant on self‐reported responses to questions, which can be seen as less reliable for measuring behavioral implementation (Kunstler et al., 2019). However, the open questions afforded us the opportunity to learn what participants thought and felt about giving mental health advice, enabling insight into why implementation might vary (Michie et al., 2005). Finally, the sample size and use of a multifactor ANOVA should also be noted as potential limitations, although our approach did match those used by other TDF investigations. In mitigation, both these factors could have contributed to the inflation of Type 1 error; however, importantly, none of our experience groups or interactions yielded significant results. In short, we can be reasonably confident in the validity of the results, albeit against an estimated 12% sample.

TDF (Michie et al., 2005) was developed to evaluate the implementation of guidelines by health care professionals, particularly where such guidelines might be seen to take the professional outside of his or her initially trained “comfort zone”. It was maybe not unsurprising given the levels of preparedness and implementation, that the practitioners in this study called for sport psychology‐specific CPD focusing on delivering mental health interventions. Therefore, we would emphasize the importance of involving the professional bodies (e.g., BASES and BPS) who govern the training and CPD in the United Kingdom to ensure that the sustainability and effectiveness of mental health promotion fits well within the codes of conducts, realms, and competencies of the applied sport psychology field.

## CONFLICT OF INTEREST STATEMENT

The authors declare that they have no conflicts of interest to disclose.
